# Association Between *CYP2C9* and *CYP2C19* Genetic Polymorphisms and Antiseizure Medication-Induced Adverse Reactions Among Peruvian Patients with Epilepsy

**DOI:** 10.3390/ph18121872

**Published:** 2025-12-09

**Authors:** Angel T. Alvarado, Felipe L. Ignacio-Cconchoy, Juan C. Espinoza-Retuerto, Roxana M. Contreras-Macazana, Luis Abel Quiñones, Jorge A. García, María R. Bendezú, Haydee Chávez, Felipe Surco-Laos, Doris Laos-Anchante, Pompeyo A. Cuba-Garcia, Elizabeth J. Melgar-Merino, Bertha Pari-Olarte, Mario Bonifaz-Hernández, José Santiago Almeida-Galindo, José Kong-Chirinos, Ricardo Pariona-Llanos, Priscilia Aguilar-Ramírez, Nelson M. Varela

**Affiliations:** 1 Center for Research in Molecular Pharmacology and 4P Medicine, VRI, San Ignacio de Loyola University, La Molina, Lima 15024, Peru; 2 Human Medicine, Faculty of Health Sciences, San Ignacio de Loyola University, La Molina, Lima 15024, Peru; 3 Internal Medicine Department, Alberto Sabogal National Hospital, Sologuren, Callao 07000, Peru; 4 Human Medicine, Faculty of Human Medicine, José Faustino Sánchez Carrión National University, Huacho 15100, Peru; 5 Neurology Department, Alberto Sabogal Sologuren National Hospital, Callao 07000, Peru; 6 Biochemistry and Immunochemistry Department, Alberto Sabogal Sologuren National Hospital, Callao 07000, Peru; 7 Faculty of Human Medicine, National University of San Marcos, Lima 15081, Peru; 8 Laboratory of Chemical Carcinogenesis and Pharmacogenetics, Department of Basic and Clinical Oncology, Faculty of Medicine, University of Chile, Santiago de Chile, Chile & Latin American Network for Implementation and Validation of Clinical Pharmacogenomics Guidelines (RELIVAF), Santiago 8350499, Chile; 9 Department of Pharmaceutical Sciences and Technology, Faculty of Chemical and Pharmaceutical Sciences, University of Chile, Santiago 8350499, Chile; 10 Faculty of Pharmacy and Biochemistry, San Luis Gonzaga National University of Ica, Ica 11004, Peru; 11 Faculty of Human Medicine, San Luis Gonzaga National University of Ica, Ica 11004, Peru; 12 General Studies, FIE, National University of San Marcos, UNMSM, Lima 15081, Peru; 13 Human Medicine, Continental University, Los Olivos, Lima 15312, Peru

**Keywords:** antiseizure medication, *CYP2C9*, *CYP2C19*, adverse drug reactions, pharmacogenomics, Peruvian population

## Abstract

**Background/Objectives**: Epilepsy is characterized by recurrent, unprovoked, self-limiting seizures of genetic, acquired, or unknown origin. It affects more than 50 million people worldwide. The prevalence in Peru is 11.9–32.1 per 1000 people. Our objective was to describe the association between *CYP2C9* and *CYP2C19* genetic polymorphisms and adverse reactions induced by antiseizure medications among Peruvian patients with epilepsy. **Methods**: A descriptive observational study was conducted on Peruvian patients with epilepsy. Non-probability, non-randomized, purposive sampling was carried out through consecutive inclusion. Genomic DNA was obtained from venous blood samples. Genotypes were determined by real-time PCR using specific TaqMan probes to identify the alleles of interest. **Results**: In total, 89 Peruvian patients with epilepsy were recruited at the Alberto Sabogal Sologuren National Hospital-ESSALUD: 45 were male (23.6 ± 10.0 years) and 44 were female (24.0 ± 12.4 years). The observed frequencies for CYP2C9*2, CYP2C9*3, CYP2C19*2, CYP2C19*3, and CYP2C19*17 were 0.034 (T allele), 0.034 (C allele), 0.14 (A allele), 0.00 (A allele), and 0.03 (T allele), respectively. Patients with intermediate and poor metabolic phenotypes of *CYP2C9* and *CYP2C19* had a significantly higher risk of adverse drug reactions (ADRs) (OR = 3.75; 95%CI: 1.32–10.69; *p* = 0.013), compared with normal metabolizers. Polytherapy was a predictor increasing the likelihood of ADRs (OR = 4.33; 95% CI: 1.46–12.80; *p* = 0.008). **Conclusions**: In this cohort of Peruvian patients with epilepsy, the reduced-function alleles CYP2C9*2, CYP2C9*3, and CYP2C19*2, associated with decreased metabolic activity, were significantly linked to an increased risk of adverse drug reactions induced by antiseizure medications. Polytherapy further heightened this risk. Collectively, these findings highlight the clinical relevance of *CYP2C9* and *CYP2C19* genotyping to enhance the safety of antiseizure pharmacotherapy in Latin American settings, where pharmacogenomic evidence remains limited.

## 1. Introduction

### 1.1. Background

Epilepsy is one of the most prevalent chronic neurological disorders worldwide, characterized by recurrent unprovoked and self-limited seizures of genetic, acquired, or unknown origin [1,2,3]. It affects more than 50 million people globally, and 80% of these cases occur in developing countries [4,5]. The prevalence of epilepsy in Peru is estimated at 11.9–32.1 per 1000 people—a range that reflects regional heterogeneity in published Peruvian studies [6,7]. Antiseizure medications (ASMs) of the aromatic type [carbamazepine (CBZ), oxcarbazepine (OXC), phenytoin (PHT), phenobarbital (PHB), levetiracetam (LEV), lamotrigine (LTG), lacosamide, primidone, rufinamide, and zonisamide] and non-aromatic type [valproic acid (VPA) and pregabalin] are used to control seizures [8,9]. These medications develop adverse drug reactions in 22 to 31% of patients with epilepsy [10]. Aromatic ASMs are associated with life-threatening type B hypersensitivity reactions, such as Stevens–Johnson syndrome (SJS), toxic epidermal necrolysis (TEN), or drug reaction with eosinophilia and systemic symptoms (DRESS) [9,11]; additionally, carbamazepine induces photosensitivity [12], and phenytoin causes gingival hyperplasia, hirsutism, and acne [12,13].

### 1.2. Genetic and Non-Genetic Factors Associated with Drug Resistance to Antiseizure Medication

It has been reported that approximately 30% of patients do not respond to treatment with ASMs [14,15]. This could be explained by drug resistance to ASMs, which may be attributed to both non-genetic factors (such as sex, age, ethnicity, seizure type, early onset of epilepsy, suboptimal dosage, lack of adherence to treatment, and alcohol abuse) and genetic factors [16]. Among the genetic factors with the most evidence for their role in drug resistance are polymorphisms in cytochrome p450 genes (*CYP2C9* and *CYP2C19*), genes that encode for UDP-glucuronosyltransferase (UGT) enzymes (*UGT1A4, UGT2B7* and *UGT2B15*) [16], and ABC genes (ATP-binding cassette) such as ABCB1 (MDR1) which encodes P-glycoprotein transporter [17,18] that is overexpressed in epileptogenic tissues [19]. Likewise, the mutation of the *SCN2A* gene [c.56 G>A (rs17183814] is known to be associated with resistance to multiple ASMs [20].

### 1.3. Pharmacogenetics and Personalized Medicine in Epilepsy

Through personalized or precision medicine, the goal is to individualize the dose from the outset of pharmacological therapy, taking into account the pharmacogenetic profile according to ethnicity, mixed race, origin, sex, and lifestyle, with the aim of maintaining plasma concentrations within the therapeutic range [21,22]. Therefore, it is essential to apply pharmacogenomics and precision medicine in epilepsy, which will facilitate individualization of the dose to optimize efficacy and minimize adverse drug reactions (ADRs) [23]. In this context, it is important to analyze the two main pharmacogenes of ASMs, such as the *CYP2C9* gene located on the long arm of chromosome 10, region 24 (10q24), which has more than 61 allelic variants and multiple suballeles. CYP2C9*1 is its wild allelic version, which, when combined, forms the CYP2C9*1/*1 genotype that predicts normal metabolism. The two most common variants with decreased enzyme activity are CYP2C9*2 (rs1799853) and CYP2C9*3 (rs1057910), while the genotypes CYP2C9*2/*3 and CYP2C9*3/*3 are predictors of poor metabolic phenotypes [24,25,26,27]. The frequency of CYP2C9*2 in Caucasians is 10–17%, in Africans 2–4%, and in Asians 6%; while the frequency of CYP2C9*3 in Caucasian populations is 6–7%, in Asians 2–11%, and it is less prevalent in African or African American ethnic groups (1%) [28]. Meanwhile, the *CYP2C19* gene is mapped to the long arm of chromosome 10, region 24.1 (10q24.1), and is highly polymorphic, with more than 25 allelic variants. The wild allele CYP2C19*1 gives rise to CYP2C19*1/*1, responsible for predicting normal metabolizers by the CYP2C19 enzyme. The CYP2C19*2 (rs4244285; c.681G>A) and CYP2C19*3 (rs4986893; c.636G>A) alleles form homozygous CYP2C19*2/*2 and CYP2C19*3/*3 genotypes, respectively, and predict poor metabolizers. The frequencies of *CYP2C19* poor metabolizers are estimated at 2% in Caucasian populations, 5% in Africans, and 15% in Asians [29,30]. The CYP2C19*17 allele (rs12248560) can account for the CYP2C19*17/*17 or CYP2C19*1/*17 genotypes, which predict rapid metabolizers [25,31,32].

Carriers with *CYP2C9* and *CYP2C19* SNPs predictive of rapid metabolism are associated with decreased drug efficacy [25,31,32]. In contrast, poor metabolizers exhibit a decrease in the safety of ASMs; for example, CYP2C9*3/*3 carriers show high plasma levels of PHT [27] and valproic acid which can induce ADRs [33]. In CYP2C19*2/*2 carriers, elevated plasma levels (69 mg/L) of PHT have been observed, leading to neurotoxicity (manifested as dizziness, nystagmus, ataxia, and excessive sedation) [34]. Therefore, it is recommended to reduce the PHT dose in intermediate and poor metabolizers [35].

### 1.4. Enzymes That Metabolize Antiseizure Medication

These genes encode their respective enzymes which metabolize various drugs in clinical use, including ASMs, such as VPA which is metabolized through phase I oxidation with the participation of *CYP2C9*, *CYP2C19*, CYP2A6, and CYP2B6, forming 4-hydroxyvalproic acid and 4-ene-VPA. Additionally, it is biotransformed by UDP-glucuronosyltransferase 2B7 (UGT2B7) and other UGTs [36,37,38,39]. Phenytoin (PHT) is biotransformed into 3′,4′-phenytoin epoxide by the action of *CYP2C9* (90%) and *CYP2C19* (10%). The metabolite then undergoes two biotransformation processes: first, 3′,4′-dihydrodiol phenytoin is generated by the action of epoxide hydrolase; subsequently, the main metabolite 5-(p-hydroxyphenyl)-5-phenylhydantoin (p-HPPH) is formed with the participation of *CYP2C9* and *CYP2C19* [40,41]. Likewise, PHB is metabolized through *CYP2C9* and *CYP2C19* into a p-hydroxyphenyl derivative [42].

### 1.5. Pharmacogenetic Studies on ASM in Peru

Despite their global clinical importance, pharmacogenomic studies on ASMs in Peru are limited compared to other countries in Latin America and around the world. Additionally, routine pharmacogenomic testing is not recommended in clinical practice, which presents a challenge for researchers to generate scientific evidence and validate results in order to propose the implementation of personalized medicine in Peru.

### 1.6. Objective

The objective of this study was to describe the association between *CYP2C9* and *CYP2C19* polymorphisms and ADRs induced by antiseizure medication in Peruvian patients with epilepsy.

## 2. Results

### 2.1. Clinical Characteristics and Medication Data of Patients

The present study describes the allelic variants of *CYP2C9* and *CYP2C19,* evaluating their association with ADRs induced by ASMs in Peruvian patients with epilepsy. The patients in the study were diagnosed by electroencephalography (*n* = 75) and by computed tomography or magnetic resonance imaging (*n* = 14) with no structural alterations. They were classified according to the International League Against Epilepsy (ILAE) into generalized seizure (*n* = 34) and focal seizure (*n* = 55). All participants had been undergoing treatment with ASMs for more than 2 years. In total, 43 had a family history of seizures (28 male, 15 female), and 55 were well controlled (no recurrence) at the time of the study, while 34 patients were uncontrolled. Those considered poor responders during the 2–6-month treatment were prescribed polytherapy and scheduled for short periods of clinical and pharmacological evaluation. A total of 45 patients were included in monotherapy with VPA, PHT, or PHB, while 44 were in polytherapy. A descriptive analysis was performed to characterize the participants, with continuous variables described as the mean and standard deviation (SD). The clinical and pharmacological characteristics of patients with epilepsy were compared by sex. Female patients received polytherapy (VPA + LTG + LEV) more frequently than males (50.0% vs. 28.9%; *p* = 0.069). Generalized epilepsy was more prevalent in women (47.7% vs. 28.9%), whereas focal seizures predominated in men (71.1% vs. 52.3%), although this did not reach significance (*p* = 0.0755). A family history of epilepsy was more common in males (62.2% vs. 34.1%; *p* = 0.0109). No differences in clinical control of epilepsy were observed between groups (*p* = 0.6657); see Table 1.

### 2.2. Genes, Allelic Variants, and Genotypes

**Figure 1:**
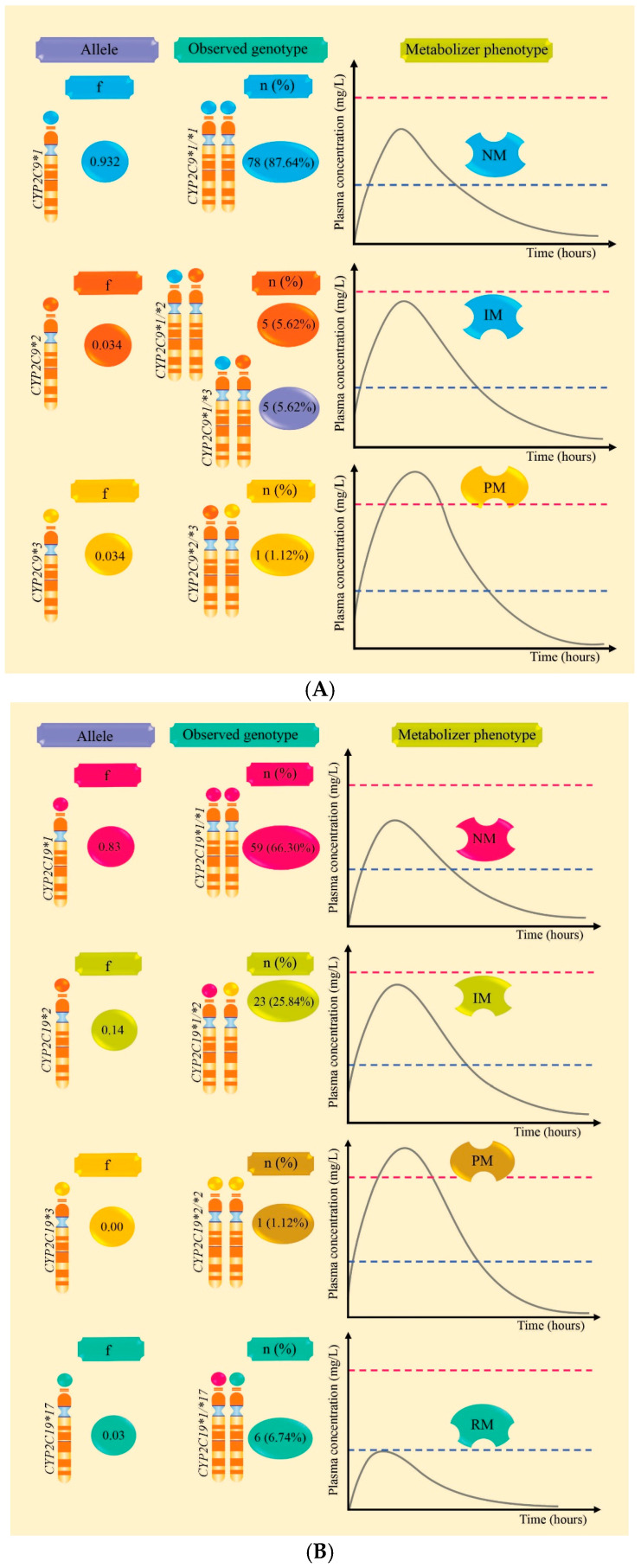
Allele frequencies, *CYP2C9/CYP2C19* genotypes, and plasma level curves according to metabolic phenotype in Peruvian patients with epilepsy are outlined. Part (**A**) presents the frequency of *CYP2C9* alleles and the percentages of genotypes that predict normal (NM), intermediate (IM), and poor (PM) metabolizers. Part (**B**) presents the frequency of *CYP2C19* alleles and the percentages of genotypes that predict normal (NM), intermediate (IM), poor (PM), and rapid metabolizers (RM).

Table 2 and Figure 1A,B report the frequencies of alleles and genotypes, revealing a higher frequency of CYP2C19*2 (0.14) compared to CYP2C9*2 and CYP2C9*3 which both had a frequency of 0.034. CYP2C19*17 was identified with a frequency of 0.03, while CYP2C19*3 was not observed. These alleles constitute the genotypes CYP2C9*1/*2, CYP2C9*1/*3, and CYP2C19*1/*2, which predict intermediate metabolizers (IM). The CYP2C9*2/*3 and CYP2C19*2/*2 genotypes predict poor metabolizers (PM), whereas the CYP2C19*1/*17 genotype predicts rapid metabolizers (RM). Additionally, CYP2C9*1/*1 and CYP2C19*1/*1 predict normal metabolizers (NM).

### 2.3. Metabolic Phenotype Related to Adverse Drug Reactions Induced by Antiseizure Medication

**Figure 2:**
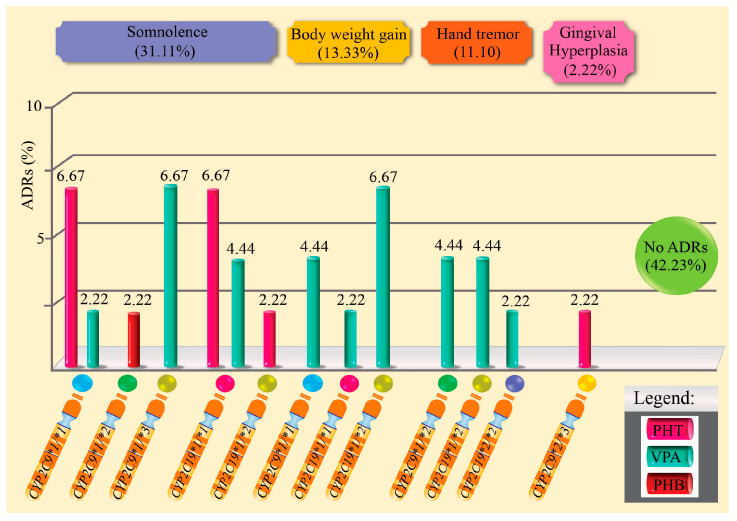
Percentage of adverse drug reactions induced by antiepileptic drugs in monotherapy and related to genotypes.

Table 3 and Figure 2 present the ADRs observed in patients with epilepsy treated with monotherapy, according to the *CYP2C9* and *CYP2C19* genotypes. Of the 45 patients evaluated, 57.8% experienced at least one ADR, with the most frequent being drowsiness (31.1%), weight gain (13.3%), and hand tremor (13.3%). Gingival hyperplasia was uncommon (4.4%), and 42.2% did not experience any ADRs. No significant associations were found between the presence of ADRs and the *CYP2C9* metabolizing phenotype (*p* = 0.156) or with the type of monotherapy drug (*p* = 0.417). However, relevant clinical trends were observed, such as a higher frequency of somnolence and weight gain in patients treated with VPA.

**Figure 3:**
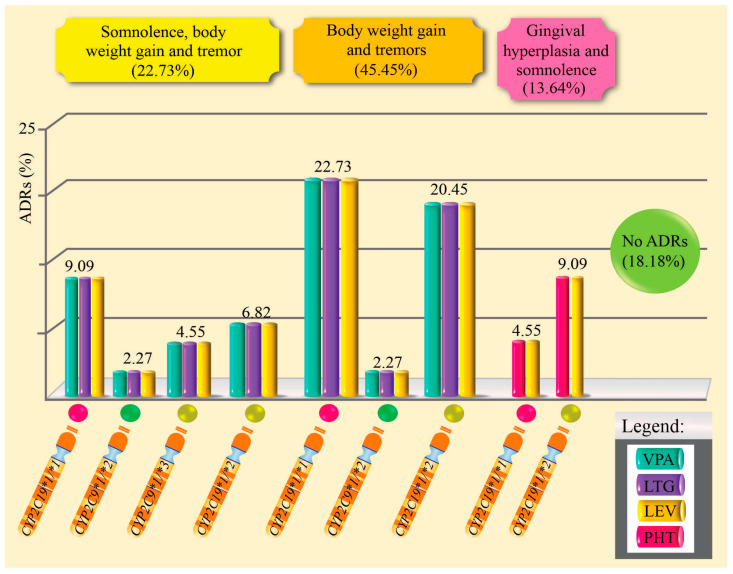
Percentage of adverse drug reactions induced by antiepileptic drugs in polytherapy and related to genotypes.

Table 4 and Figure 3 show the distribution of ADRs in patients with epilepsy treated with combination therapy, according to the *CYP2C9* and *CYP2C19* gene genotypes, their respective metabolic phenotypes, and the pharmacological regimen used. Of the total of 44 patients, 81.8% (*n* = 36) presented at least one ADR. The most frequent adverse reactions were weight gain and hand tremors (45.5%), followed by somnolence (22.7%), and to a lesser extent, gingival hyperplasia combined with somnolence (13.6%). Only 18.2% of patients (*n* = 8) did not experience any ADRs. When analyzing the metabolic phenotype of the *CYP2C9* gene, it was observed that intermediate metabolizers (IM) had a higher proportion of ADRs compared to normal metabolizers (NM). Specifically, 97.0% of patients with the IM phenotype experienced at least one ADR, while in the NM group, the frequency was 73.7%. However, this difference was not statistically significant (*p* = 0.904; chi-square test). Regarding the type of polytherapy used, it was found that the VPA + LTG + LEV combination was associated with a higher number of adverse drug reactions compared to the PHT + LEV regimen. This difference was statistically significant (*p* = 0.0015; Fisher’s exact test), suggesting a possible influence of the type of pharmacological combination on the occurrence of adverse drug reactions.

In the multivariate analysis, two independent variables showed statistically significant associations with the presence of ADRs. Intermediate and PM patients for *CYP2C9* and *CYP2C19* were found to have a 3.75 times higher probability of experiencing ADRs compared to normal metabolizer patients (*p* = 0.013). Additionally, it was shown that the likelihood of presenting ADRs is 4.33 times greater in patients treated with polytherapy (*p* = 0.008). In contrast, the type of seizure (focal vs. generalized) was not significantly associated with the risk of ADRs (*p* = 0.379); see Table 5.

## 3. Discussion

### 3.1. Interpretation of Findings and Comparison with Literature

The dose of ASMs administered in male and female epilepsy patients influences absorption. Previous studies have shown that there are several factors that influence absorption. For instance, the study by Martinez and Amidon (2002) indicates that gastric emptying time, intestinal transit time, pH of the absorption medium, blood flow at the absorption site, presystemic metabolism, gastrointestinal content, and disease state influence the intestinal absorption of drugs [43]. In addition to patient factors, age and sex [44], the physicochemical properties (solubility and pKa) [43], drug polymorphism (crystalline form and amorphous), and the characteristics of the pharmaceutical form (excipients, formulation, and technological process) also influence the absorption and bioavailability of drugs [45]. These differences may affect pharmacotherapeutic efficacy and increase the risk of ADRs.

The allele frequencies of CYP2C9*2, CYP2C9*3, CYP2C19*2, CYP2C19*3, and CYP2C19*17 were 3.4%, 3.4%, 14%, 0%, and 3%, respectively, among patients with epilepsy in the present study. Meanwhile, the frequencies of the CYP2C9*1/*2, CYP2C9*1/*3, and CYP2C19*1/*2 genotypes were 5.62%, 5.62%, and 25.84%, respectively; these genotypes predict IM. The frequency of the *CYP2C9* polymorphism, as well as that of CYP2C9*1/*2, is similar to previous studies conducted in the Peruvian population, which found an allelic frequency of 4.6% for CYP2C9*2 [25] and a genotype frequency of 4.3% for CYP2C9*1/*2 [46]. This confirms that these variants are present in the study population regardless of health condition, indicating that they express enzymes with lower activity compared to the wild-type CYP2C9*1/*1 allele. Additionally, a frequency for CYP2C9*3 of 6.2% [25] to 6.25% [47] was found, suggesting that some frequencies remain consistent while others vary within the Peruvian population.

No studies were found concerning Peruvian patients with epilepsy. In this regard, when compared with other previously published studies on other populations, notable differences are observed. Lakhan et al. (2011) reported frequencies CYP2C9*2, CYP2C9*3, and CYP2C19*2 of 8%, 23.4%, and 49%, respectively, in Indian patients [48]. Makowska et al. (2021) found in Polish children with epilepsy two alleles, CYP2C9*2 and CYP2C19*2, accounting for 26.3% and 30.5%, respectively [49]. In another study by Fohner et al. (2020), which involved patients with epilepsy treated with phenytoin, the population comprised 21 patients (5%) who self-identified as Asian; 18 (5%) as Black; 29 (8%) as Hispanic Caucasian; and 308 (81%) as non-Hispanic Caucasian [50]. In this population, the frequencies of CYP2C9*2 and CYP2C9*3 were observed to be 12.0% and 4.7%, respectively, with 20% for CYP2C19*17, 17% for CYP2C19*2, and 1% for CYP2C19*3. Likewise, Song et al. (2022) reported in patients with epilepsy from Yunnan Province, China, that the frequencies of CYP2C19*2 and CYP2C19*3 were 33.1% and 3%, respectively, among patients receiving VPA treatment [51]. These differences are likely influenced by the tricontinental and Latin American ancestry of Peruvians, as well as the internal migration that occurs from the Andes and jungle to the Peruvian coast. This highlights the need to conduct analytical observational studies (cases/controls and cohorts) and randomized clinical trials (RCTs) in pharmacogenetics with a larger number of patients [52,53,54].

This study also identified a carrier of CYP2C9*2/3 (1.12%) and another of CYP2C192/*2 (1.12%), both of which predict PM; these patients were treated with PHT and VPA, respectively. Due to the decreased metabolism of drugs in intermediate metabolizers or the lack of metabolism in PM, serum levels may exceed minimum toxic concentrations, leading to ADRs. Previous studies have established the minimum effective plasma concentrations and the minimum toxic plasma concentrations for ASMs as follows: VPA 50–100 mg/L [39,55], PHT 10–20 mg/L [56,57], and PHB 10–40 mg/L [58]. However, other factors that influence the increase in plasma levels must also be considered, such as diet type, drug–nutrient interaction, volume of distribution, plasma protein binding [59], enzyme inhibitors [60], drug interactions in polytherapy [61], liver and kidney dysfunction, advanced age, and other variables [62].

This study evaluated the relationship between pharmacogenomic and clinical factors with the occurrence of ADRs in patients with epilepsy, using an internally validated logistic regression model. In total, 1000 bootstrap resampling iterations were performed to generate data sets and the area under the curve (AUC) was calculated, yielding an average of 0.722 (95% CI: 0.683–0.747) which supports the robustness and adaptability of the model in the available sample despite the moderate sample size. The area under the curve was 0.747, indicating the good discriminatory capacity of the model. McFadden’s Pseudo R^2^ was 0.13, suggesting the reasonable explanatory power of the model in contexts with multiple clinical factors. The Akaike Information Criterion (AIC) value was 102.99, analyzed as a comparative metric for alternative models. The confusion matrix revealed an overall accuracy of 69.7%, with a sensitivity of 80.6% (the ability to correctly identify patients with ADRs) and a specificity of 44.4% (the ability to identify those who did not experience ADRs). Therefore, intermediate and poor metabolizers for *CYP2C9* and *CYP2C19* were found to be associated with ADRs. Additionally, it was revealed that polytherapy is a significant predictors of ADRs, this result aligns with previously published prospective studies indicating an association between ASM polytherapy and a higher risk of ADRs [63,64,65,66].

The ADRs observed in the intermediate and poor metabolizer patients in the present study were not severe, in contrast to those described in previous research. PHT is associated with gingival hyperplasia, epigastric pain, drowsiness, lethargy, headache, confusion [67], immune thrombocytopenia [68], and risk of liver injury [69]. Additionally, tremor, weight gain, hair loss [48], metabolic acidosis, hyperammonemia, hepatotoxicity, thrombocytopenia, and gastrointestinal disorders have been reported for VPA [70].

It is worth noting that several investigations have associated genotypes with ADRs. The study by Orsini et al. (2018) found an association between CYP2C9*2 and CYP2C9*3 with the onset of non-alcoholic fatty liver disease and severe VPA-induced hyperammonemia [71]. Monostory et al. (2019) observed decreased metabolism and increased plasma levels and ADRs due to VPA in carriers of CYP2C9*2 and CYP2C9*3 [72]. Similarly, Song et al. (2022) reported high plasma levels of VPA in carriers of CYP2C19*2 and CYP2C19*3, associated with the risk of ADRs [51]. Shnayder et al. (2023) described that CYP2C9*2 and CYP2C9*3 are associated with decreased metabolism and increased plasma levels of VPA [73]. Iannaccone et al. (2021) observed that carriers of CYP2C9*2 and CYP2C9*3 have increased plasma levels of VPA compared to the wild-type CYP2C9*1 allele [33]. Garg et al. (2022) found a significant association between CYP2C9*3 and the risk of gingival hyperplasia and PHT-induced rash [74]. Recently, Milosavljevic et al. (2024) reviewed the significant association of *CYP2C9* and *CYP2C19* genes with increased plasma levels of PHT and VPA [75]. Six carriers of CYP2C19*17 (rs12248560, g.-806C>T) were also found, a genotype which increases the transcription of genes encoding enzymes with increased metabolic activity [76]. In patients carrying the CYP2C19*1/*17 genotypes, which predict rapid metabolizers, no adverse reactions were observed in the present study. In rapid metabolizers, the minimum effective plasma concentration is not achieved which may contribute to resistance or therapeutic failure.

### 3.2. Clinical Importance

These findings reinforce the clinical importance of pharmacogenetic testing as a tool for identifying patients with rapid, intermediate, and poor metabolic phenotypes and for proposing personalized doses. This approach prioritizes monotherapy when feasible or rational combinations based on the genetic profile, plasma drug levels, and the patient’s clinical characteristics. The Dutch Pharmacogenetics Working Group (DPWG) has recommended that in *CYP2C9* intermediate and poor metabolizers, the daily dose of PHT should be titrated according to clinical effect and plasma levels after 7–10 days [35].

### 3.3. Strengths and Limitations

The limitations of this study include the sample size of patients with epilepsy (*n* = 89). No relevant genes associated with adverse reactions to ASMs were identified, such as *ABCB1* which encodes the P-glycoprotein (also called the ABCB1 transporter); *ABCC2,* which encodes the MRP2 transporter; *UGT2B7*, which encodes the enzyme that conjugates VPA; and *SCN2A*, which encodes the alpha subunit of the sodium channel. Additionally, this study did not include carbamazepine and the genes encoding the enzymes CYP3A4 and CYP3A5 that metabolize this drug. Plasma levels of ASMs were not evaluated. All of these limitations will be addressed in future research by our research group.

Furthermore, we have not studied the Human Leukocyte Antigen (HLA) alleles that are common in populations outside of the Peruvian mestizo population. HLA-B*15:02 is common in populations from East Asia (6.9%), Oceania (5.4%), and South/Central Asia (4.6%) [77,78,79], with a frequency of 2.5% in Koreans, less than 1% in Japanese, Caucasian, Hispanic/South American, and Middle Eastern populations; it has not been observed in Africans [80]. Meanwhile, HLA-A*31:01 is observed in Japanese (8%), Hispanic/South American (6%), South Korean (5%), and Caucasian (3%) populations, as well as in South and Central Asian (2%) populations [80]. HLA-B*15:02 is associated with Stevens–Johnson syndrome (SJS) and toxic epidermal necrolysis (TEN), while HLA-A*31:01 is linked to an increased risk of drug reaction with eosinophilia and systemic symptoms (DRESS), as well as SJS/TEN induced by aromatic antiepileptic drugs [11,80]. Despite the modest sample size, this cohort provides baseline pharmacogenomic data from Peruvian patients with epilepsy, supporting the design of larger multicentric studies.

However, this observational study is the first to describe the allelic variants of *CYP2C9* and *CYP2C19* associated with adverse reactions in patients with epilepsy from a reference hospital in the coastal area of Callao, Peru. Additionally, these findings could serve as important scientific evidence to enhance the potential for prescribing medications at personalized doses that ensure efficacy while minimizing ADRs. Future studies integrating multi-gene panels and therapeutic-drug monitoring could further validate these associations in larger Latin American cohorts.

## 4. Materials and Methods

### 4.1. Study Design, Population, and Type of Sampling

A descriptive, observational, prospective, and cross-sectional study was designed. The study population consisted of 89 Peruvian patients diagnosed with generalized and focal seizures who attended the Neurology Service of the Alberto Sabogal Sologuren National Hospital (HNASS) of the Social Health Security (ESSALUD), between November 2023 and October 2024. A total of 45 (50.56%) male patients (range = 18–85 years) and 44 (49.44%) female patients (range = 18–79 years) were included. After signing their informed consent, they were called volunteer patients.

Sampling was non-probabilistic, convenient, non-random, intentional, and involved consecutive inclusion of patients with epilepsy according to the inclusion and exclusion criteria.

### 4.2. Inclusion and Exclusion Criteria

Patients attending the Neurology Service of HNASS for routine clinical monitoring were invited to participate in the study. A total of 115 patients were informed about the purpose and importance of the study, and only 89 were selected according to the following inclusion criteria: patients diagnosed with epilepsy who were receiving ASMs as monotherapy (VPA, PHT, and PHB) or polytherapy (VPA + LTG + LEV or PHT + LEV) for more than two years; controlled patients with a good response to treatment; patients on polytherapy who were considered poor responders; adherence to treatment (taking medication at the indicated times and with 250 mL of water); a commitment not to self-medicate and to communicate if they required any medication other than for their condition; being Peruvian by birth and residing in the Province of Callao; being over 18 years of age; and a commitment to donate 5 mL of blood for SNP screening in CYP2C9 and CYP2C19, as well as signing informed consent before starting the study.

Fifteen patients were excluded due to lack of therapeutic follow-up and non-adherence to treatment. Eleven were excluded for being treated with carbamazepine or other medications not classified as ASMs, as well as pregnant patients and anyone who did not meet the inclusion criteria.

### 4.3. Obtaining Genomic DNA

Genomic DNA (gDNA) was obtained from the buffy coat of blood samples using a standard manufacturer’s protocol. Subsequently, gDNA was quantified by spectrophotometry using the Denovix^®^ equipment (model DS-11, FX, Spectrophotometer™ Series, Wilmington, DE, USA). Samples were analyzed at two absorbance wavelengths, 260/280 nm and 260/230 nm, and were considered suitable when the absorbance ratios were equal to or greater than 1.7. gDNA samples were stored at −20 °C until further analysis.

### 4.4. Genotypic Analysis

*CYP2C9* and *CYP2C19* genotypes were determined by real-time PCR (RT-PCR) with the identification of allelic variants using TaqMan probes for genotyping assays (Thermo Fischer Scientific Inc., Waltham, MA, USA), which are capable of discriminating the identified SNPs CYP2C9*2 (rs1799853), CYP2C9*3 (rs1057910), CYP2C19*2 (rs4244285), CYP2C19*3 (rs4986893), and CYP2C19*17 (rs12248560). According to the standard protocol, the reaction mix consisted of 30 ng of gDNA, Genotyping Master Mix™ (Cat. No. 4371355), TaqMan SNP Genotyping Assay™ (with corresponding probes and primers from Thermo Fisher Scientific Inc.), and nuclease-free molecular-biology-grade water (HyPure, HyClone™Thermo Fischer Scientific Inc., Waltham, MA, USA), sufficient for a final reaction volume of 10 μL. Context-specific sequences are listed in Table 6.

For amplification, the Stratagene Mx3000P™ equipment (Agilent Technologies, Waldbronn, Germany) was used; the program included an initial cycle of enzyme activation (AmpliTaq Gold^®^ Thermo Fischer Scientific Inc., Waltham, MA, USA), and denaturation at 95 °C for 10 min, followed by 50 cycles of 15 s of denaturation at 95 °C and 90 s of annealing and extension at 60 °C.

### 4.5. Statistical Analysis

To analyze the association between the seizure type and sex, as well as to evaluate sex differences in valproic acid pharmacotherapy, Fisher’s exact test was applied considering a value of *p* < 0.05 as significant. Phenytoin and phenobarbital were evaluated using the Kruskal–Wallis test to identify significant differences in the doses administered associated with sex and the type of antiseizure medication. The multivariate model identified factors associated with adverse drug reactions, considering independent variables selected by expert judgment: *CYP2C9* metabolic phenotype, *CYP2C19* phenotype, type of pharmacotherapy, and seizure type. The results were expressed as an odds ratio (OR) with 95% confidence intervals (95% CIs), with significance set at *p* < 0.05. Statistical analysis was performed using the Python language (version 3.9), with the pandas libraries for data processing, stats models for multivariate logistic regression models, and scikit-learn for results validation.

### 4.6. Ethical Considerations

The study was conducted in accordance with the Declaration of Helsinki. The Research Ethics Committee of the Alberto Sabogal Sologuren National Hospital-ESSALUD in Callao approved the protocol and informed consent for the study as a minimal-risk investigation, using blood samples from routine clinical practice, through Memorandum No. 098-CIEI-OIyD-GRPS-ESSALUD-2023. The subjects signed informed consent before participating and were referred to as volunteer patients. Each volunteer was assigned a code to ensure anonymity and confidentiality.

## 5. Conclusions

In this cohort of Peruvian patients with epilepsy, the reduced-function alleles CYP2C9*2, CYP2C9*3, and CYP2C19*2, associated with decreased metabolic activity, were significantly linked to an increased risk of adverse drug reactions induced by antiseizure medications. Polytherapy further heightened this risk.

Collectively, these findings highlight the clinical relevance of CYP2C9 and CYP2C19 genotyping to enhance the safety of antiseizure pharmacotherapy in Latin American settings, where pharmacogenomic evidence remains limited.

## Figures and Tables

**Table 1:** Anthropometric, clinical, and medication characteristics of patients with epilepsy.

Variable	Male (*n* 45, 50.56%)			Female (*n* 44, 49.44%)			*p*-Value
	Mean ± SD		Range	Mean ± SD		Range	
Age (years)	23.6 ± 10.0		18–85	24.0 ± 12.4		18–79	0.5912 *
Monotherapy	*n* = 28 (62.20%)	Mean ± SD	Range	*n* = 17 (37.70%)	Mean ± SD	Range	
VPA (dose mg/day)	18 (40.0%)	1722 ± 491.76	1000–2500	9 (20.5%)	1388.89 ± 333.33	1000–2000	0.483 *
PHT (dose mg/day)	9 (20.0%)	300	300	8 (18.2%)	300	300	
PHB (dose mg/day)	1 (2.2%)	300	300	0 (0.0%)	0	0	
Polytherapy	*n* = 17 (37.80)			*n* = 27 (61.30)			
VPA (dose mg/day)	13 (28.9%)	1500	1500	22 (50.0%)	1500	1500	0.069 **
LTG (dose mg/day)		100	100		100	100	
LEV (dose mg/day)		3000	3000		3000	3000	
PHT (dose mg/day)	4 (8.9%)	300	300	5 (11.3%)	300	300	0.972 **
LEV (dose mg/day)		3000	3000		3000	3000	
Seizure type							
Generalized	13 (28.90%)			21 (47.70%)			0.0755 **
Focal	32 (71.10%)			23 (52.30%)			
With a family history of seizure	28 (62.2%)			15 (34.10%)			0.0109 *
No family history of seizure	17 (37.8%)			29 (65.90%)			
Controlled (without recurrence)	29 (64.4%)			26 (59.1%)			0.6657 *
Not controlled	16 (35.6%)			18 (40.9%)			

VPA: valproic acid; PHT: phenytoin; PHB: phenobarbital; LTG: lamotrigine; LEV: levetiracetam; SD: Standard deviation; * Mann–Whitney U test *p*-value; ** Pearson’s chi-squared. *p* < 0.05 is considered significant.

**Table 2:** Allele and genotype frequencies of *CYP2C9* and *CYP2C19* in patients with epilepsy.

Gene	Allele			Observed Genotype		Metabolizer Phenotype
	Type	*n*	f	Type	*n* (%)	
*CYP2C9*	*1	166	0.932	*1/*1	78 (87.64)	NM
	*2	6	0.034	*1/* 2	5 (5.62)	IM
	*3	6	0.034	*1/*3	5 (5.62)	IM
				*2/*3	1 (1.12)	PM
		178	1.00		89 (100%)	
*CYP2C19*	*1	147	0.83	*1/*1	59 (66.30)	NM
	*2	25	0.14	*1/*2	23 (25.84)	IM
	*3	0	0.00	*2/*2	1 (1.12)	PM
	*17	6	0.03	*1/*17	6 (6.74)	RM
		178	1.00		89 (100%)	

NM: normal metabolizer; IM: intermediate metabolizer; PM: poor metabolizer; RM: rapid metabolizer.

**Table 3:** Genotypes and metabolic phenotypes related to adverse reactions induced by monotherapy with antiseizure medication.

Genotype	Phenotype	ASM	ADRs Observed in the Study				No ADRs *n* (%)	Total *n* (%)
			Somnolence *n* (%)	Body Weight Gain *n* (%)	Hand Tremor *n* (%)	Gingival Hyperplasia *n* (%)		
CYP2C9*1/*1	NM	VPA	1 (2.22)	2 (4.44)	0 (0.00)	0 (0.00)	4 (8.89)	7 (15.56)
		PHT	3 (6.67)	0 (0.00)	0 (0.00)	0 (0.00)	3 (6.67)	6 (13.33)
CYP2C19*1/*1	NM	VPA	2 (4.44)	1 (2.22)	0 (0.00)	0 (0.00)	3 (6.67)	6 (13.33)
		PHT	3 (6.67)	0 (0.00)	0 (0.00)	0 (0.00)	2 (4.44)	5 (11.11)
CYP2C9*1/*2	IM	VPA	0 (0.00)	0 (0.00)	2 (4.44)	0 (0.00)	0 (0.00)	2 (4.44)
		PHB	1 (2.22)	0 (0.00)	0 (0.00)	0 (0.00)	0 (0.00)	1 (2.22)
CYP2C9*1/*3	IM	VPA	3 (6.67)	0 (0.00)	0 (0.00)	0 (0.00)	0 (0.00)	3 (6.67)
CYP2C9*2/*3	PM	PHT	0 (0.00)	0 (0.00)	0 (0.00)	1 (2.22)	0 (0.00)	1 (2.22)
CYP2C19*1/*2	IM	VPA	0 (0.00)	3 (6.67)	2 (4.44)	0 (0.00)	0 (0.00)	5 (11.11)
		PHT	1 (2.22)	0 (0.00)	0 (0.00)	0 (0.00)	1 (2.22)	2 (4.44)
CYP2C19*2/*2	PM	VPA	0 (0.00)	0 (0.00)	1 (2.22)	0 (0.00)	0 (0.00)	1 (2.22)
CYP2C19*1/*17	RM	VPA	0 (0.00)	0 (0.00)	0 (0.00)	0 (0.00)	3 (6.67)	3 (6.67)
		PHT	0 (0.00)	0 (0.00)	0 (0.00)	0 (0.00)	3 (6.67)	3 (6.67)
Total:			14 (31.11)	6 (13.33)	5 (11.10)	1 (2.22)	19 (42.23)	45 (100%)

ASM: antiseizure medication; ADRs: adverse drug reactions; chi-square test for the presence of ADRs according to *CYP2C9* phenotype (*p* = 0.156); chi-square test for the presence of ADRs according to ASM in monotherapy (*p* = 0.417). *p* < 0.05 is considered significant.

**Table 4:** Genotypes and metabolic phenotypes related to adverse reactions induced by polytherapy with antiseizure medication.

Genotype	Phenotype	ASM	ADRs Observed in the Study			No ADRs *n* (%)	Total *n* (%)
			Somnolence, Body Weight Gain, and Tremor *n* (%)	Body Weight Gain and Tremors *n* (%)	Gingival Hyperplasia and Somnolence *n* (%)		
CYP2C19*1/*1	NM	VPA, LTG, LEV	4 (9.09)	10 (22.73)	0 (0.00)	5 (11.36)	19 (43.18)
		PHT, LEV	0 (0.00)	0 (0.00)	2 (4.55)	3 (6.82)	5 (11.36)
CYP2C9*1/*2	IM	VPA, LTG, LEV	1 (2.27)	1 (2.27)	0 (0.00)	0 (0.00)	2 (4.55)
CYP2C9*1/*3	IM	VPA, LTG, LEV	2 (4.55)	0 (0.00)	0 (0.00)	0 (0.00)	2 (4.55)
CYP2C19*1/*2	IM	VPA, LTG, LEV	3 (6.82)	9 (20.45)	0 (0.00)	0 (0.00)	12 (27.27)
		PHT, LEV	0 (0.00)	0 (0.00)	4 (9.09)	0(0.00)	4 (9.09)
Total:			10 (22.73)	20 (45.45)	6 (13.64)	8 (18.18)	44 (100%)

ASM: antiseizure medication; ADRs: adverse drug reactions; Pearson’s chi-square: comparison of ADRs presence by metabolic phenotype (NM vs. IM) (*p* = 0.904); Fisher’s exact test: comparison of ADRs presence between combination therapy regimens VPA + LTG + LEV vs. PHT + LEV (*p* = 0.0015). *p* < 0.05 is considered significant.

**Table 5:** Multivariate logistic regression analysis between adverse reactions and independent variables.

Independent Variables/Reference Category	OR	95%CI	*p*-Value
Metabolic phenotype			
NM (Referring)	-	-	-
IM and PM	3.75	1.32–10.69	0.013 *
Type of pharmacotherapy			
Monotherapy (Referring)	-	-	-
Polytherapy	4.33	1.46–12.80	0.008 *
Seizure type			
Focal (Referring)	-	-	-
Generalized	1.62	0.55–4.77	0.379

OR: odds ratio; CI: confidence intervals. Metabolic phenotype includes carriers of *CYP2C9* and *CYP2C19*; NM: normal metabolizer; IM: intermediate metabolizer; PM: poor metabolizer. AIC: Akaike Information Criterion = 102.99; McFadden’s Pseudo R^2^ = 0.13; significance: *p* < 0.05 (*).

**Table 6:** Sequence for identification of single nucleotide polymorphisms of *CYP2C9* and *CYP2C19* genes.

Allele (dbSNP)	Context Sequence TaqMan SNP Genotyping Assay™
CYP2C9*2 (rs1799853)	GATGGGGAAGAGGACATTGAGGAC[C/T] GTGTTCAAGAGGAAGCCCGCTGCCT
CYP2C9*3 (rs1057910)	TGTGGTGCACGAGGTCCAGAGATAC[C/A] TTGACCTTCTCCCCACCAGCCTGCC
CYP2C19*2 (rs4244285)	TTCCCACTATCATTGATTATTTCCC[A/G] GGAACCCATAACAAATTACTTAAAA
CYP2C19*3 (rs4986893)	ACATCAGGATTGTAAGCACCCCCTG[A/G] ATCCAGGTAAGGCCAAGTTTTTTGC
CYP2C19*17 (rs12248560)	AAATTTGTGTCTTCTGTTCTCAAAG[C/T] ATCTCTGATGTAAGAGATAATGCGC

*dbSNP*: single nucleotide polymorphism database.

## Data Availability

The original contributions presented in this study are included in the article. Further inquiries can be directed to the corresponding author.

## Abbreviations

ADRs	Adverse drug reactions
ASMs	Antiseizure medications
AUC	Area under the curve
AIC	Akaike Information Criterion
gDNA	Genomic DNA
ILAE	International League Against Epilepsy
HNASS	Alberto Sabogal Sologuren National Hospital
ESSALUD	Social Health Security
95% CI	95% confidence intervals
OR	Odds ratio
DRESS	Drug reaction with eosinophilia and systemic symptoms
SJS	Stevens–Johnson syndrome
TEN	Toxic epidermal necrolysis
IM	Intermediate metabolizers
NM	Normal metabolizers
PM	Poor metabolizers
RM	Rapid metabolizers
LEV	Levetiracetam
LTG	Lamotrigine
PHT	Phenytoin
PHB	Phenobarbital
VPA	Valproic acid
